# Simultaneous Determination of Ciprofloxacin and Ofloxacin in Animal Tissues with the Use of Capillary Electrophoresis with Transient Pseudo-Isotachophoresis

**DOI:** 10.3390/molecules26226931

**Published:** 2021-11-17

**Authors:** Izabella Kośka, Krystian Purgat, Rafał Głowacki, Paweł Kubalczyk

**Affiliations:** 1 Department of Environmental Chemistry, Faculty of Chemistry, University of Lodz, Pomorska 163, 90-236 Lodz, Poland; krystian.purgat@edu.uni.lodz.pl (K.P.); rafal.glowacki@chemia.uni.lodz.pl (R.G.); 2 Doctoral School of Exact and Natural Sciences, University of Lodz, Banacha 12/16, 90-237 Lodz, Poland

**Keywords:** capillary electrophoresis, transient pseudo-isotachophoresis, extraction, antibiotics, ciprofloxacin, ofloxacin

## Abstract

We have developed a precise and accurate method for the determination of ciprofloxacin and ofloxacin in meat tissues. Our method utilizes capillary electrophoresis with a transient pseudo-isotachophoresis mechanism and liquid–liquid extraction during sample preparation. For our experiment, a meat tissue sample was homogenized in pH 7.00 phosphate buffer at a ratio of 1:10 (tissue mass: buffer volume; g/mL). The extraction of each sample was carried out twice for 15 min with 600 µL of a mixture of dichloromethane and acetonitrile at a 2:1 volume ratio. We then conducted the electrophoretic separation at a voltage of 16 kV and a temperature of 25 °C using a background electrolyte of 0.1 mol/L phosphate–borate (pH 8.40). We used the UV detection at 288 nm. The experimentally determined LOQs for ciprofloxacin and ofloxacin were 0.27 ppm (0.8 nmol/g tissue) and 0.11 ppm (0.3 nmol/g tissue), respectively. The calibration curves exhibited linearity over the tested concentration range of 2 to 10 nmol/g tissue for both analytes. The relative standard deviation of the determination did not exceed 15%, and the recovery was in the range of 85–115%. We used the method to analyze various meat tissues for their ciprofloxacin and ofloxacin contents.

## 1. Introduction

Fluoroquinolones (FQLs) are derivatives of nalidixic acid and belong to a class of synthetic antibiotics. These compounds are commonly used in treating respiratory diseases, urinary tract infections, and gastric disorders. FQLs possess powerful bactericidal properties. They inhibit the transcription and replication of bacterial DNA by cutting bacterial DNA in the gyrase DNA and type IV topoisomerase enzymatic complexes [1,2]. This bactericidal activity is observed against both Gram-positive and Gram-negative bacteria. In addition, they show excellent bioavailability due to rapid absorption following oral administration and a broad spectrum of activity [1,3]. FQLs are used not only for treatment, but also for the prevention of infectious diseases in humans and animals. Our approach addresses the risk of overprescribing this group of antibiotics and releasing excessive amounts of these compounds into the environment through urine and feces. Excessive consumption of FQLs can cause many problems and lead to allergic reactions, diseases, and even cancer. Another problem related to the use of antibiotics is the effect of antibiotic resistance due to the formation of antibiotic-resistant bacteria. This is one of the most serious problems today [3,4].

Therefore, the development of new, sensitive and selective analytical methods for the determination of FQL residues in food and the environment is of the utmost importance [5,6]. These actions should enable the optimal dose of the relevant drugs to be judged, thus minimizing treatment time in order to prevent the negative effects of antibiotic use [2].

This paper describes how we developed our method for detecting ciprofloxacin (Cpx) and ofloxacin (Ofx) in animal tissues. Several HPLC-UV methods enable the determination of these compounds in various matrices, e.g., in human plasma and urine [2,7]. Methods based on HPLC-MS for FQLs’ determination in wastewater [8,9] or animal products [10,11] are also described in the literature. We also detail CE methods that allow the identification of some FQLs in human urine [12] and animal products [13]. In this work, we decided to use CE due to its advantages over HPLC, such as high efficiency for separation, a short analysis time, and, importantly, lower consumption of reagents and samples. Such a development is desirable from both an environmental standpoint and in the interests of green chemistry. Due to the limitations of this technique, i.e., a relatively high detection limit compared to other, more sophisticated techniques, some methods of sample purification and concentration were applied [6,13,14,15,16,17].

## 2. Results and Discussion

### 2.1. Optimization of Electrophoretic Conditions

#### 2.1.1. Optimization of BGE Concentration and pH of BGE

The first optimized parameter was the BGE concentration. We investigated the influence of the BGE concentration on the height of the analytical signals, peak size repeatability, and resolution. To test these correlations, we selected the following concentrations of phosphate–borate buffer (pH 8.40): 0.01 mol/L, 0.02 mol/L, 0.05 mol/L, 0.1 mol/L, 0.15 mol/L, 0.175 mol/L, and 0.2 mol/L. In the case of BGE concentrations in the range of 0.01–0.05 mol/L, no signals from the analytes appeared. Since 0.1 mol/L phosphate–borate buffer demonstrated the highest signals, we selected this concentration of BGE for further studies. Subsequently, we checked the effect of pH of BGE on the peak height, size repeatability, and resolution. We studied the following pHs of the 0.1 mol/L phosphate–borate buffer: 8.00, 8.20, 8.40, 8.60, and 8.80. The peaks from Cpx and Ofx did not separate satisfactorily when the buffers were at pH 8.00 and 8.20, while from pH 8.40 upwards, these peaks were isolated to the baseline. In Figure S1, one can observe that the highest analytical signals were obtained for pH 8.40. Therefore, we selected as BGE for further experiments the phosphate–borate buffer with a concentration of 0.1 mol/L consisting of 0.1 mol/L NaH_2_PO_4_ and 0.1 mol/L Na_2_B_4_O_7_ adjusted to pH 8.40.

#### 2.1.2. Selection of the Sample Volume Introduced to the Capillary

We investigated the influence of the sample volume introduced into the capillary on the height of the analytical signal. For this, we introduced the sample into the capillary hydrodynamically by alternating the amount of pressure and the length of time applied. We introduced the sample for 10 s at a pressure of 5 mbar, then at 10 mbar, 20 mbar, and 30 mbar, and a pressure of 10 mbar, 20 mbar, 30 mbar for 20 s, and 60 mbar for 30 s. The introduction of a larger sample volume probably overloaded the capillary because it resulted in an unstable current. The highest analytical signals were observed in the case of sample introduction by applying a pressure of 60 mbar for 30 s (217 nL, 7.6% total volume of capillary). We introduced the sample into the capillary using this method during further experiments.

#### 2.1.3. Optimization of Capillary Temperature and Separation Voltage

**Figure 1:**
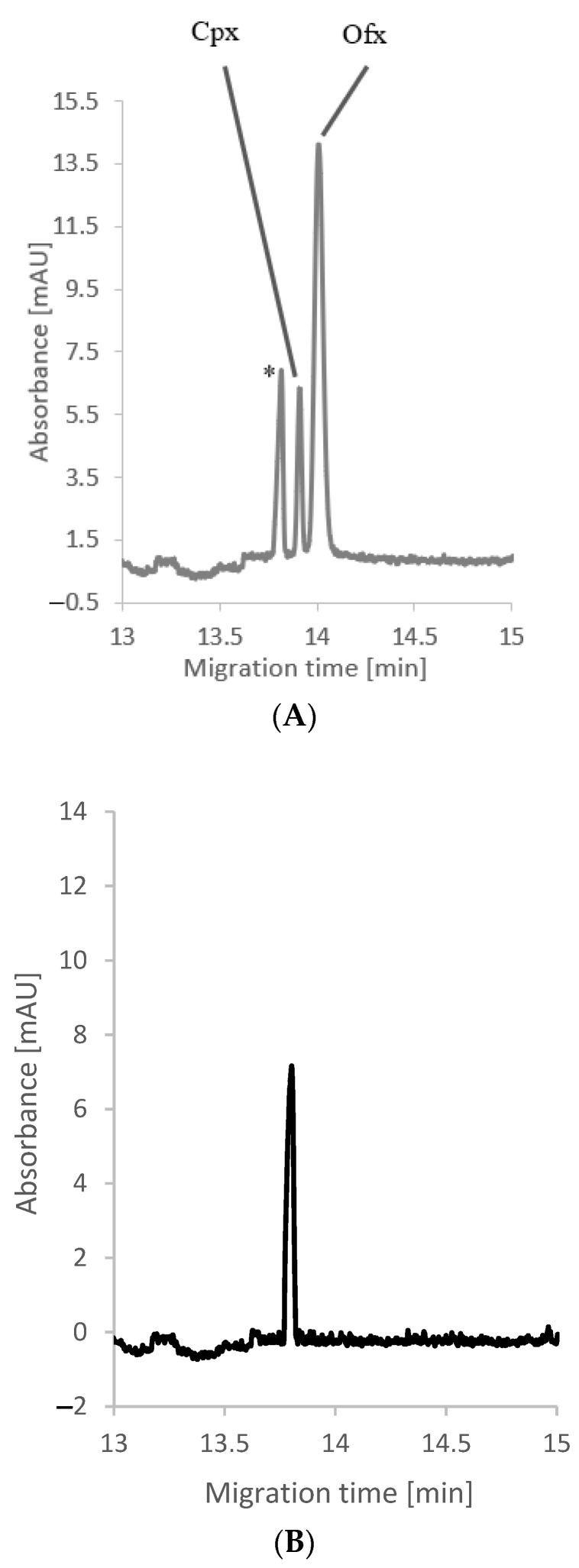
(**A**) Representative electropherogram obtained for meat tissue spiked with Cpx and Ofx (final concentration 1.4 ppm (4 nmol/g tissue)). * The peak corresponds to the non-identified component of the sample. (**B**) Representative electropherogram obtained for blank meat tissue.

Having identified that, among the temperatures between 20 and 27 °C, the highest signals occur at 25 °C, we chose a voltage of 16 kV since the sample stacking method relates to the mechanism of transient pseudo-isotachophoresis. This value did not cause excessive Joule heating. It ensured the signals were satisfactory, but above all, the Cpx and Ofx peaks separated to the baseline within 15 min, as shown in Figure 1A. It is commonly known that meat tissue has a complex matrix containing cells, minerals, salts, etc. Since SDME is not entirely a specific method, other substances besides analytes were extracted and visible on the electropherogram. An unknown peak that migrates at 13.8 min appears in Figure 1B. It is related to the substance(s) present in the sample and, thankfully, does not interfere with the analytes.

#### 2.1.4. Sample Stacking by Transient Pseudo-Isotachophoresis

A notable limitation of the CE separation methods compared to the HPLC is that the concentration sensitivity is inferior when used with current commercial instrumentation equipped with UV-Vis absorption detectors. In our experiment, we applied transient pseudo-isotachophoresis to achieve sample concentration directly on the capillary before the separation step took place. The meat tissue sample evaporated to dryness after homogenization and extraction. We dissolved it in a mixture of acetonitrile and 0.01 mol/L NaOH (1:3 *v*/*v*) and then hydrodynamically injected it into the capillary as a long plug. Once we turned on the voltage, small cations (sodium or others) made rapid movement due to high mobility and the presence of low conductivity acetonitrile, slowing down at the interface of the BGE. Because of the increased field in that area vacated by the small cations, analytes (Ofx, Cpx) in the region move fast, while those in front or close to the inorganic cations slow down and remain behind, giving rise to stacking. The rationale for labelling this method of stacking transient pseudo-isotachophoresis is that small inorganic cations act as leading ions while acetonitrile operates as a pseudo-terminator.

### 2.2. Optimization of Extraction Procedure

#### 2.2.1. Selection of Buffer pH for Sample Preparation

The choice of pH for the homogenization buffer was an invaluable parameter in the development of this sample preparation procedure, as the pH of the sample at this stage determines the equilibrium state of the extraction and therefore the extraction’s efficiency. During the selection of the sample pH for extraction, knowing the pKa of the analytes is valuable. The pKa values of Cpx are 6.00 for the carboxylic acid group and 8.80 due to the nitrogen on the piperazinyl ring, while the pKa values of Ofx are 6.10 and 8.28, respectively [13]. Considering the pKa values mentioned above, the correlation between the height of the analytical signals and the pH of 0.2 mol/L phosphate buffer was tested in the range from 5.00 to 9.00. As evident in Figure S2, the level of the signals increases until the sample pH of 7.00, when the peak heights reach their maximum values and then decrease. Therefore, we chose a 0.2 mol/L phosphate buffer that we adjusted to pH 7.00 by mixing 0.2 mol/L H_3_PO_4_ with 0.2 mol/L Na_3_PO_4_ for the sample preparation.

#### 2.2.2. Tissue to Buffer Ratio

The following ratios of animal tissue mass to buffer volume were checked for homogenization: 1:2, 1:4, 1:6, 1:8, 1:10, and 1:15 (g/mL). In Figure S3, the peak heights increased with rising sample dilution until a 1:10 (g/mL) dilution. Further dilution of the sample did not increase the extraction efficiency. Thus, we selected the 1:10 (g/mL) ratio of tissue mass to buffer volume.

#### 2.2.3. Selection of Organic Solvent

After optimizing the homogenization parameters, we checked the influence of the type of organic phase on the extraction efficiency. While selecting organic solvents, we carefully chose those immiscible with the donor phase to ensure the phases were separated satisfactorily following the extraction process. Therefore, following solvents were investigated: dichloromethane, chloroform, ethyl acetate, hexane, and dichloromethane mixtures with chloroform, ethyl acetate, and acetonitrile in the following ratios: 4:1, 2:1, 1:1, 1:2, and 1:4 (*v*/*v*). The dichloromethane and acetonitrile mixture gave the highest peaks at a volumetric ratio of 2:1. Therefore, this organic mixture was used as acceptor phase to perform the Cpx and Ofx extraction in this experiment.

#### 2.2.4. Selection of Organic Solvent Volume

Subsequently, we checked the influence of organic phase volume on the extraction efficiency. For this purpose, the extraction took place with various portions of the organic solvent, i.e., 200 µL, 400 µL, 600 µL, 800 µL, and 1000 µL. We observed no increase in the extraction efficiency for volumes greater than 600 µL, as per Figure S4. Hence, we extracted samples with a 600 µL portion of the organic phase in further studies.

#### 2.2.5. Optimization of Extraction Time

Another optimized parameter was extraction time, which we tested in the range from 5 to 60 min. Figure S5 shows that there is no increase in peak height and no improvement in repeatability for extraction times longer than 15 min. This lull indicates that the equilibration between the sample and organic phase takes place within 15 min, so we chose this as the extraction time.

#### 2.2.6. The Number of Extractions

We checked whether multiple extractions of the same sample would affect the extraction efficiency. To test this, we performed extractions once, twice, and thrice. However, we achieved reliable repeatability when we extracted the same tissue sample twice (Figure S6). Therefore, we performed the sample extraction twice.

### 2.3. Calibration and Other Validation Data

The developed method for simultaneous Ofx and Cpx determination in meat tissues was validated in accordance with the criteria set for the analysis of biological samples [18]. We determined the limits of detection (LOD) and quantification (LOQ) experimentally and then chose an analyte concentration for which the signal is three times higher than the baseline noise as the LOD. Likewise, we identified the LOQ as an analyte concentration for which the signal is nine times higher than the baseline noise. The LOD value (0.15 mAU) evaluated for Cpx was 99.4 ppb (0.3 nmol/g) and 36.1 ppb (0.1 nmol/g) for Ofx. The LOQ values (0.45 mAU) were 0.27 ppm (0.8 nmol/g) for Cpx and 0.11 ppm (0.3 nmol/g) for Ofx, respectively. The LOQs of the developed method are lower than for CE-DAD procedure [19] (for Cpx 0.098 mg/kg (0.3 µmol/g) and for Ofx 0.090 mg/kg (0.25 µmol/g)), similar to HPLC-FLD procedure [20] (for Ofx LOQ is: 40 µg/kg (0.1 nmol/g)) and for LC-MS/MS procedure [21] (for Cpx LOQ is 125 µg/kg (0.38 nmol/g)), but higher than for LC–MS/MS methods [22] (Cpx 22 ng/g, and Ofx 24 ng/g (0.07 nmol/g)) and [23] (Cpx and Ofx 5 µg/kg (0.015 nmol/g)). We constructed five-point calibration curves for Cpx and Ofx in meat tissues in the concentration range from 2 to 10 nmol/g tissue. Each series was performed in triplicate. The obtained calibration curves (Figure S7) showed linearity in the tested concentration range. The linear correlation coefficient square (R^2^) for Cpx and Ofx was 0.9988 and 0.9987, respectively. The equation of the calibration curve for Cpx was y = (1.0816 ± 0.0223)x − (0.7933 ± 0.1484), and for Ofx y = (5.1558 ± 0.1041)x − (1.6816 ± 0.6905). The RSD of the Cpx calibration curve points ranged from 10.9 to 5.2%, and for Ofx, the recovery ranged from 96.4% to 101.9%. These values are consistent with the analysis criteria for biological samples [19]. In the next stage, we examined intraday and interday precision. For this purpose, we selected three concentrations from the range of the calibration curve. The meat tissue samples were prepared for the calibration curve and analyzed. We selected the first concentration (3 nmol/g) from the beginning of the calibration curve range, the next concentration (5 nmol/g) represented the center of the calibration curve, and the final concentration was at the end of the calibration curve (9 nmol/g). Both the precision (RSDs ≤ 11.8%) and the accuracy (in the range of 85–115%) are at a satisfactory level. Therefore, in our opinion, our method for the determination of Cpx and Ofx can be used in the routine analysis of meat tissues. All the validation data are available in Table 1.

The procedure for tissue sample preparation described in this paper is simple and not time consuming. The overall time needed to prepare and analyze the meat sample for Cpx and Ofx content equals 55 min (during this time, seven samples can be prepared simultaneously due to the seven places in the vortex). This analysis time is shorter in the magnetic solid-phase extraction HPLC-MS [24] (72 min), single drop microextraction CE [12] (61 min), and excitation–emission matrix with an alternating normalization-weighted error (EEM-ANWE) [25] (155 min) procedure.

It is commonly known that the use of an internal standard can improve the method repeatability. The best choice for internal standard is a substance similar to an analyte in terms of its physicochemical, electrophoretic, and spectroscopic properties (e.g., isotopically labelled analytes). It must be absent from a sample at the same time. Unfortunately, we do not have access to these compounds. On the other hand, our results show that the method has acceptable repeatability with RSDs < 12%. Therefore, all the experiments were successful without the use of the internal standard.

### 2.4. Determination of Cpx and Ofx in Meat Tissue

Once we developed and validated our method, we used it to identify Cpx and Ofx in meat tissues such as chicken liver, kidneys, and duck and turkey liver. No traces of analytes were detected in any of tissue samples. This may indicate that the concentration of these compounds is below the LOD of the method, or these compounds are not present in the tested meat. This would be extremely positive news because, in Poland, the use of fluoroquinolones as feed additives is prohibited, so this is an encouraging finding, as it would indicate that local meat producers are complying with this regulation.

## 3. Materials and Methods

### 3.1. Apparatus

We performed all our experiments with an apparatus for the CE Agilent 7100 CE System (Waldbronn, Germany) coupled with an absorbance diode-array detector. This apparatus is equipped with an automatic injector. The fused-silica capillary we used (Polymicro Technologies, Phoenix, AZ, USA) is characterized by effective length of 56 cm, total length of 64.5 cm and inner diameter of 75 µm. We attributed the peaks to the analytes by comparing the spectra and the migration times on the electropherograms of the standard and biological samples. We used Agilent ChemStation software for quantitative analysis and to measure the height and area of the peaks. We used a pH-meter (Mettler-Toledo, Columbus, OH, USA) to adjust the pH of the solutions and thermostat (Grant, UK) to evaporate the organic solvent. Finally, we utilized a Millipore Milli-Q-RG System (Waterford, Ireland) to deionize the water.

### 3.2. Chemicals

The standards of analytes, i.e., Cpx (C_17_H_18_FN_3_O_3_) and Ofx (C_18_H_20_FN_3_O_4_) were sourced from Sigma Aldrich (Saint Louis, MO, USA). We purchased sodium tetraborate decahydrate (Na_2_B_4_O_7_·10 H_2_O) from Sigma (Steinheim, Germany) and boric acid (H_3_BO_3_), methanol (CH_3_OH), and dichloromethane (CH_2_Cl_2_) from J.T. Baker (Deventer, The Netherlands). Sodium chloride (NaCl) and sodium hydroxide (NaOH) were from POCH (Gliwice, Poland). Hexane was obtained from Lab-Scan (Dublin, Ireland). Last, we acquired chloroform (CHCl_3_), ethyl acetate (C_4_H_8_O_2_), and toluene (C_7_H_8_) from Chempur (Piekary Śląskie, Poland).

### 3.3. Capillary Preconditioning

The new capillary was conditioned with 1 mol/L NaOH solution for 20 min, followed by 20 min with 0.1 mol/L NaOH solution, 2 min with water, and 30 min with background electrolyte (BGE). Every morning, the capillary was rinsed for 5 min with 1 mol/L NaOH solution, 20 min with 0.1 mol/L NaOH solution, 2 min with water, and 30 min with BGE solution. After finishing work, we rinsed the capillary with water for 20 min and then left the capillary ends overnight in vials with water.

### 3.4. Electrophoretic Conditions

Sample concentration by transient pseudo-isotachophoresis, also called acetonitrile-salts stacking, was invented and introduced by Shihabi [26,27,28] for the analysis of samples that themselves contain a high concentration of salts. A larger injection volume of a sample (up to 30% of capillary capacity) than in the classical capillary zone electrophoresis mode (1–3% of capillary capacity) is possible with this technique. In isotachophoresis, the crucial feature is that all analytes move at the same velocity when reaching a steady state. However, the analyte speed depends on the mobility of the leading ion and its concentration. A terminating ion, which determines the high field strength, is essential to keep the analyte in the band and prevent its diffusion. Thus, acetonitrile functions as a terminating pseudo-ion by supplying the high field strength required to push or speed up the analytes’ migration without being an ion itself [15]. Transient pseudo-isotachophoresis works successfully as an analytes concentration technique for determining several compounds [29,30,31,32,33] in genuine samples of high salinity.

We used 0.1 mol/L, pH 8.40 phosphate–borate buffer as the BGE (0.1 mol/L NaH_2_PO_4_/ 0.1 mol/L Na_2_B_4_O_7_). We used hydrodynamic injection of the sample at 60 mbar for 30 s. The samples were analyzed at a temperature of 25 °C, while the applied voltage for separation was 16 kV. We performed UV-Vis detection at a wavelength of 288 nm for both FQLs.

### 3.5. Sample Collection and Preparation

We purchased animal tissues (liver, kidneys) in local stores, then portioned and stored them at a reduced temperature (−20 °C). We placed 0.2 g of tissue in a 3 mL polypropylene tube with 2 mL of 0.2 mol/L phosphate buffer (pH 7.00) and homogenized. This mixture was centrifuged at 13,680× *g* (12,000 rpm) for 10 min and 400 µL of the homogenate was transferred to a 2 mL Eppendorf tube. We added 600 µL of the organic phase (2:1 mixture of dichloromethane and acetonitrile) to the homogenate and shook it for 15 min at 3000 rpm. After extraction, we centrifuged the sample at 13,680× *g* (12,000 rpm) for 5 min. Next, we transferred 600 µL of the organic solvent (lower phase) to another Eppendorf tube and evaporated to dryness. We repeated the extraction process twice for each sample. After evaporation, we dissolved the residue in 50 µL of the mixture of acetonitrile and 0.01 mol/L NaOH (3:1, *v*/*v*) and analyzed by CE.

### 3.6. Calibration of the Method

Stock solutions of each analyte, which were then used to prepare the calibration solutions, were prepared at a concentration of 3 µmol/mL in 0.1 mol/L HCl. We fixed three series of Cpx and Ofx solutions with concentrations in the range of 2–10 nmol/g tissue for calibration. These solutions were prepared according to the following procedure: hen’s liver was homogenized with 0.2 mol/L, pH 7.00 phosphate buffer, at a ratio of 1:10 (g/mL) in a 3 mL polypropylene tube. The homogenate was centrifuged at 13,680× *g* (12,000 rpm) for 10 min, while 400 µL was transferred to the Eppendorf tube. Then, increasing amounts of the standard working solutions were added to the homogenate to obtain concentrations: 2 nmol/g tissue, 4 nmol/g tissue, 6 nmol/g tissue, 8 nmol/g tissue, and 10 nmol/g tissue. Next, 600 µL of a mixture of dichloromethane and acetonitrile in the volume ratio 2:1 was added, the tube was vigorously shaken at 3000 rpm for 15 min, and centrifuged at 13,680× *g* (12,000 rpm) for 5 min. After the phases’ separation, 600 µL of the organic phase was transferred to the Eppendorf tube and evaporated to dryness. This extraction procedure was repeated twice for each sample. After evaporation, the residue was dissolved in 50 µL of acetonitrile and 0.01 mol/L NaOH (3:1, *v*/*v*) and analyzed. After the experiments, we plotted the peak areas of Cpx and Ofx versus corresponding concentrations and fitted the curves using least-squares linear regression analysis.

## 4. Conclusions

We have developed a quick, easy, and cost-effective analytical procedure for identifying Cpx and Ofx in animal meat tissues. We developed our method based on sample clean up and analytes concentration by duplicate liquid–liquid sample extraction, online sample stacking by transient pseudo-isotachophoresis, capillary electrophoretic separation, and UV-Vis detection. The sample preparation procedure compares favorably against other methods of determining these compounds in animal tissues both in time saved and the efficiency of analysis. Our procedure is quick and easy to perform. In addition, our model produces high sensitivity and precision as well as good linearity. Considering all the advantages of our method, we believe it can usefully serve in the routine analysis of meat for Cpx and Ofx content. This effort is essential to avoid consuming these compounds with food.

## Figures and Tables

**Table 1:** Validation data.

Added * [nmol/g Tissue]	Intra-Day			Inter-Day		
	Found ± SD [nmol/g Tissue]	RSD [%]	Accuracy [%]	Found ± SD [nmol/g Tissue]	RSD [%]	Accuracy [%]
**Ciprofloxacin**						
3.00	3.14 ± 0.37	11.79	104.57	3.14 ± 0.33	10.63	104.57
5.00	4.92 ± 0.56	11.32	98.49	5.17 ± 0.40	7.79	103.42
9.00	8.87 ± 0.09	1.04	98.54	8.62 ± 0.53	6.10	95.81
**Ofloxacin**						
3.00	2.71 ± 0.18	6.56	90.39	2.72 ± 0.31	11.33	90.61
5.00	4.74 ± 0.53	11.15	94.71	4.80 ± 0.46	9.57	96.00
9.00	8.66 ± 0.97	11.15	96.22	8.21 ± 0.79	9.60	91.19

* n = 3.

## Data Availability

CE data are available from the authors.

## Supplementary Materials

The following are available online, Figure S1: The relationship between the peak height and the pH oh the background electrolyte, Figure S2: The relationship between the peak height and the pH oh the buffer for sample preparation, Figure S3: The relationship between the peak height and the ratio of tissue to 0.2 mol/L phosphate buffer, Figure S4: The relationship between the peak height and the volume of organic phase, Figure S5: The relationship between the peak height and the time of extraction, Figure S6: The relationship between the peak height and the times of extraction, Figure S7: Calibration curves.

## Sample Availability

Meat tissue samples are not available from the authors.
