# Supplementary material for: Simultaneous Determination of Ciprofloxacin and Ofloxacin in Animal Tissues with the Use of Capillary Electrophoresis with Transient Pseudo-Isotachophoresis
Source: Molecules. 2021 Nov 17;26(22):6931. doi: 10.3390/molecules26226931 (PMC8618939; doi:10.3390/molecules26226931)
Supplement: Supplementary file 1 [file molecules-26-06931-s001.zip › molecules-1448455-supplementary.pdf]

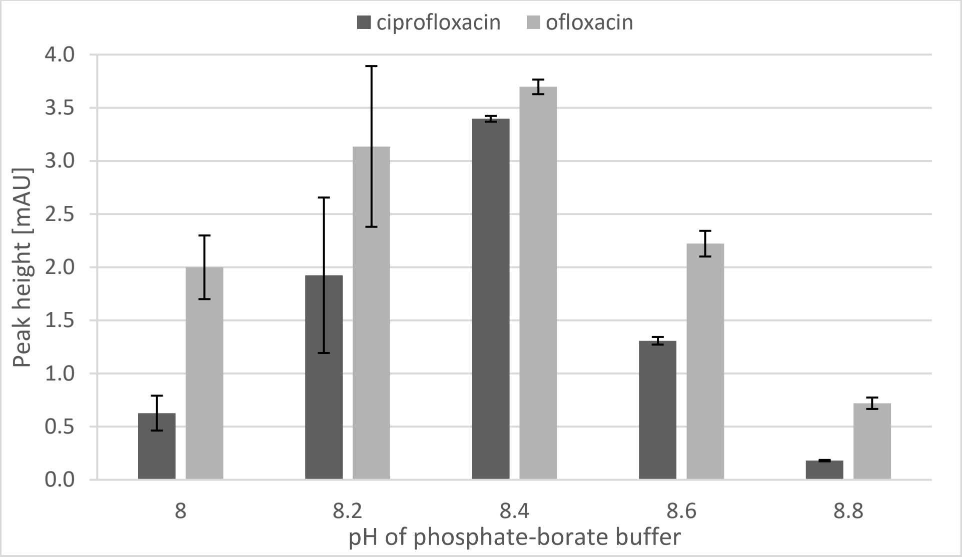

Figure S1. The relationship between the peak height and the pH oh the background electrolyte .

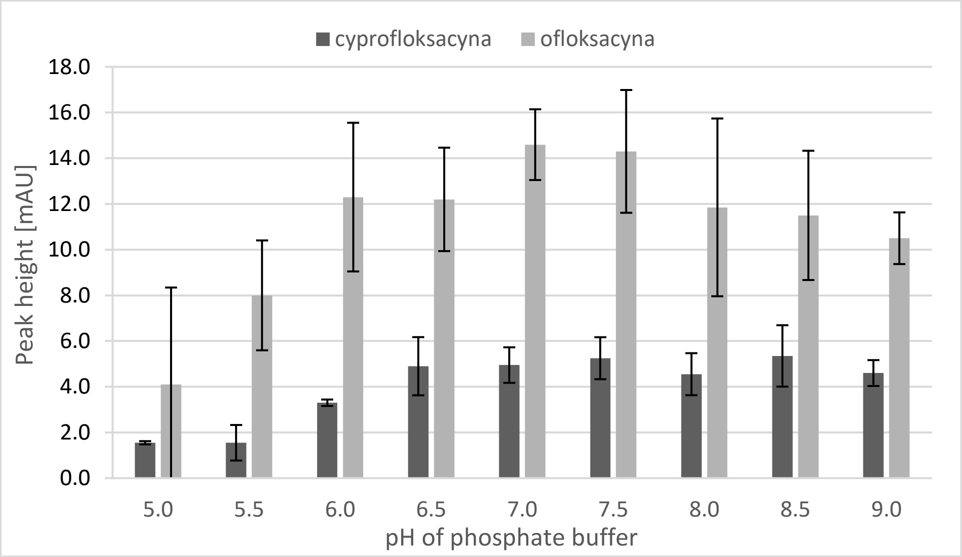

Figure S2. The relationship between the peak height and the pH of the buffer for sample preparation.

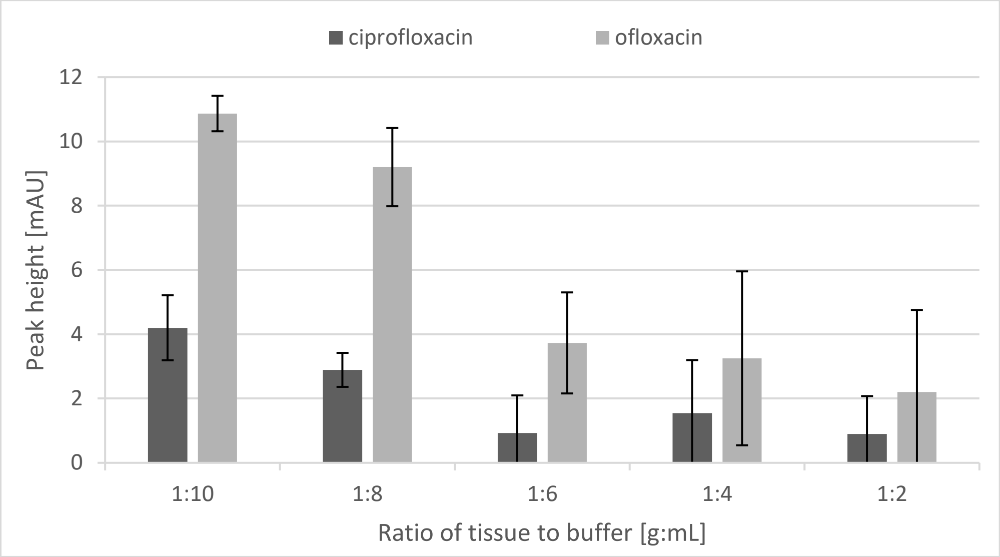

Figure S3. The relationship between the peak height and the ratio of tissue to 0.2 mol/L phosphate buffer.

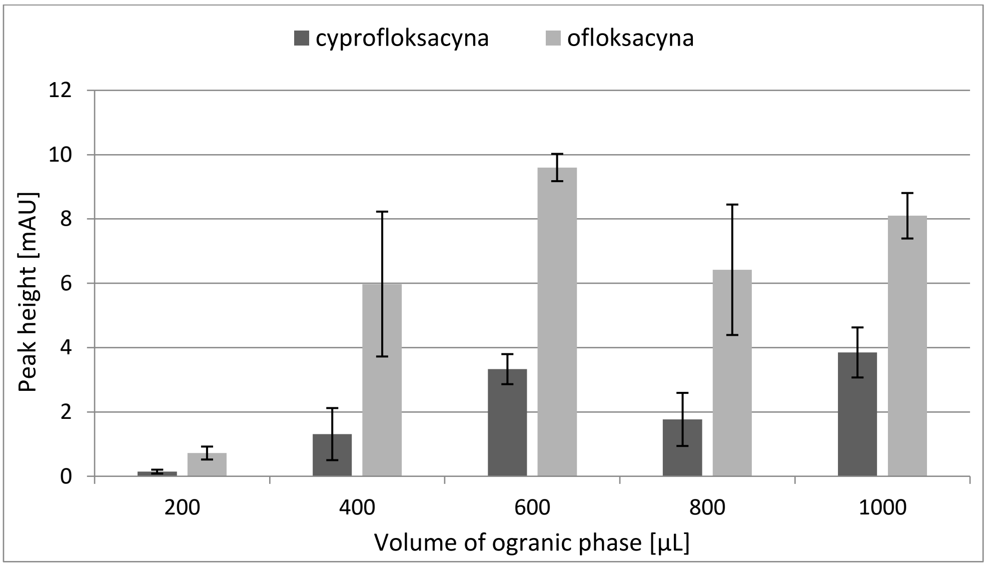

Figure S4. The relationship between the peak height and the volume of organic phase.

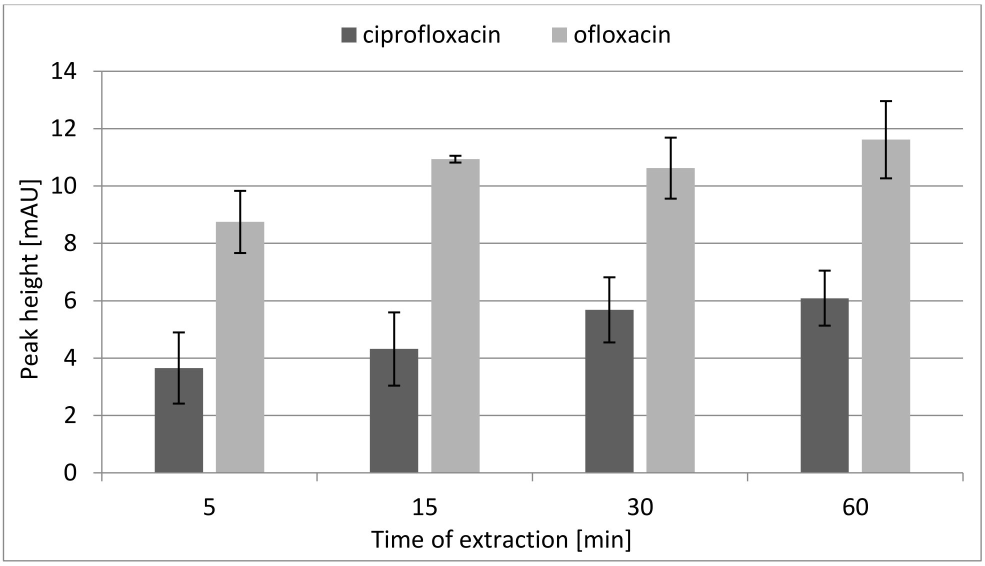

Figure S5. The relationship between the peak height and the time of extraction.

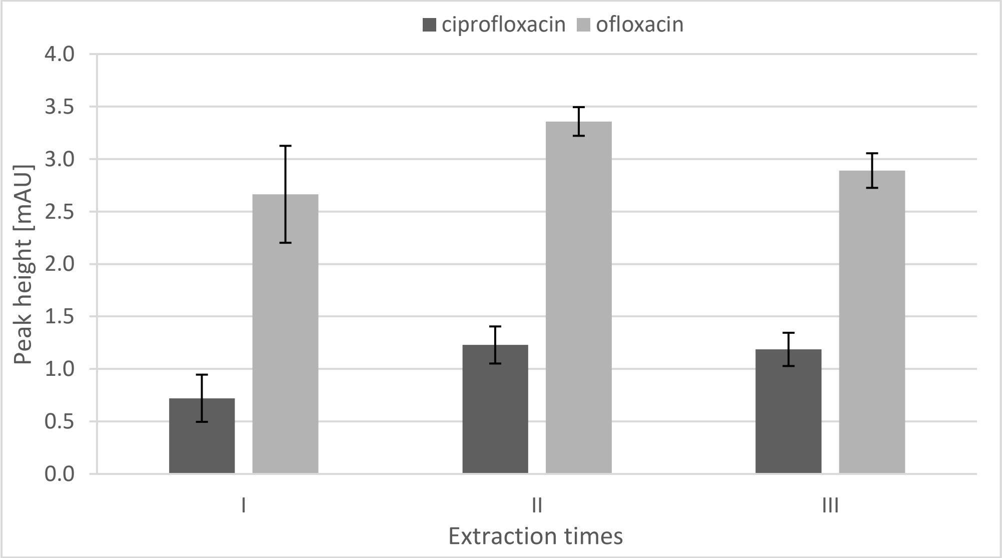

Figure S6. The relationship between the peak height and the times of extraction.

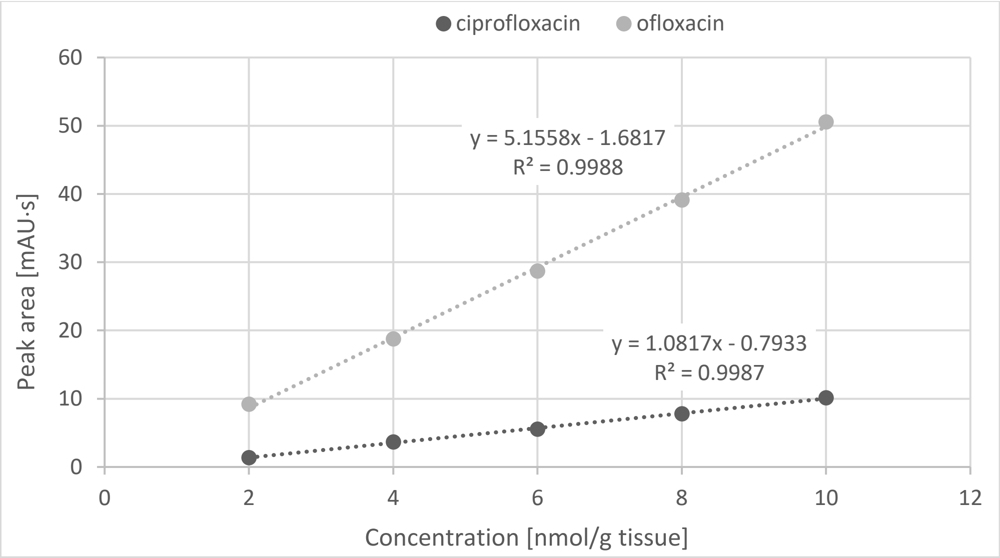

Figure S7. Calibration curves.
